# Differences in engineers’ brain activity when CAD modelling from isometric and orthographic projections

**DOI:** 10.1038/s41598-023-36823-9

**Published:** 2023-06-15

**Authors:** Fanika Lukačević, Niccolò Becattini, Marija Majda Perišić, Stanko Škec

**Affiliations:** 1 Department of Design, Faculty of Mechanical Engineering and Naval Architecture, University of Zagreb, Ivana Lučića 5, 10000 Zagreb, Croatia; 2 Department of Mechanical Engineering, Politecnico di Milano, Milan, Italy

**Keywords:** Neuroscience, Engineering

## Abstract

A way of presenting information in visual representations of technical systems influences the progress and the outcome of the engineering design process. Consequently, improving the means by and through which information is utilised during the process is one suggested approach to advancing engineering design. Engineers’ interaction with visual representations of technical systems is mainly visual and virtual. Although such interactions are cognitively complex, little is known about cognition (mental information processing) underlying the utilisation of design information during engineering design. To narrow the research gap, this study explores how visual representations of technical systems affect engineers’ brain activity while generating computer-aided design (CAD) models based on them. More precisely, the brain activity of 20 engineers is captured and analysed using electroencephalography (EEG) during the visuospatially-intensive design tasks of CAD modelling in two conditions; when technical systems are presented with orthographic and isometric projections in technical drawings. The results imply the sensitivity of engineers’ brain activity in CAD modelling to the visual representation from which a technical system is interpreted. In particular, significant differences are found in theta, alpha, and beta task-related power (TRP) over the cortex when interpreting the technical drawings and CAD modelling from them. Furthermore, the results reveal significant differences in theta and alpha TRP when considering the individual electrodes, the cortical hemispheres, and the cortical areas. In particular, theta TRP over the right hemisphere and the frontal area seems essential in distinguishing neurocognitive responses to the orthographic and isometric projections. As such, the conducted exploratory study sets the foundations for exploring engineers’ brain activity while performing visuospatially-intensive design tasks, whose segments are relatable to the aspects of visuospatial thinking. Future work will explore brain activity in other design activities that are highly visuospatial, with a larger sample size and an EEG device of a higher spatial resolution.

## Introduction

Engineering design is a process in which stakeholders (e.g. industrial designers, mechanical engineers, customers) constantly generate and use design information^1^. The process is divided into design activities, during which requirements and descriptions of a design problem (input information) are transformed into technical documentation that takes the form of representations and descriptions of the desired technical systems (output information)^2^. In current engineering practice, visual design representations come as two-dimensional (2D) and three-dimensional (3D), depending on the number of presented dimensions (height, width, and depth) and the medium used to present them^3^. Common examples of 2D visual design representations are sketches and technical drawings (orthographic, oblique or axonometric)^4^, while computer-aided design (CAD) models and physical prototypes are popular 3D visual design representations that engineers use^5^. In addition, visual design representations may be recorded and stored using various media to allow the stakeholders to see, review, criticise, evaluate, and revise them^6^. The presented study focuses on the graphically-based iconic/pictorial representations as visual records of the technical systems’ characteristics and properties (i.e., visual design representations)^2,7^.

Previous studies argued that a way of presenting information in visual design representations influences the progress and the outcome of the process^8^. Consequently, one suggested approach to enhancing engineering design is improving the means by and through which information is utilised during the process^9^. Engineers’ interaction with visual design representations is mainly visual (information is presented visually) and virtual (situated in virtual environments of CAD and engineers’ minds)^6,10^. The cognitive complexity of such interactions positions the neurocognitive perspective of utilising design information at the core of further development of design representations and computer support in design^9,11^. Previous studies shed light on the effects that the representation type (e.g. technical drawing as a 2D or CAD model as a 3D representation), media (e.g. paper, monitor screen, virtual reality) and tools (e.g. CAD tools) may have on engineers’ interaction with design information by employing protocol analysis as a dominant method for observing cognitive behaviour (e.g. Goldschmidt^8,12^ or Suwa and Tversky^13^)^14^. Still, little is known about cognition (mental information processing) underlying the utilisation of visual design representations during engineering design. In particular, neurocognition underlying design activities has been rarely studied so far, primarily due to the lack of reliable methods for its monitoring and measuring^11,15^.

Electroencephalography (EEG) is a neuroimaging method proposed for studying the neurocognitive perspective of design cognition. The primary reason for EEG’s applicability to design cognition research is its non-invasiveness which allows continuous-in-time monitoring of brain activity during design activities^14^. Recent EEG studies in design aimed to describe a neurocognitive perspective of different aspects of design thinking, primarily creativity. EEG studies explored the neurocognitive differences between design tasks^16^ (such as open and constrained tasks) or activities^17^ (e.g. decision-making, ideation, sketching). Moreover, EEG proved to help highlight differences in design neurocognition according to the previous experience of engineers^18^ (e.g. novice and experts), background^19^ (e.g. mechanical engineers, industrial designers, and architects), gender (male/female), etc. At the same time, only a few EEG studies focused on generating visual design representations, CAD modelling, and engineers’ interaction with virtual models and environments^20^. Some findings may be extracted from the studies that captured engineers’ EEG signals while solving design tasks; however, only a few of them segmented design tasks into epochs relatable to the visual processing of information (e.g. Nguyen et al.^21^).

Previous EEG studies from other fields (primarily cognitive psychology) often investigated the visual processing of information through standardized tests (such as the mental rotation test) related to the aspects of visuospatial thinking (such as spatial visualization and mental rotation)^22–27^. The results of these previous studies imply that the higher power in the theta and beta frequency bands reflect the cognitive processing of visuospatial information^22–25^. For example, Liu et al*.*^26^ reported increased (compared to the baseline) theta and beta band power in the frontal cortical area when solving the mental rotation task. In addition, an increase in theta band power over the frontal cortical area has been related to attention allocation during the task^28^. On the contrary, it has been shown that alpha band power is often reduced during visuospatial information processing and decreases with increasing processing demands^27,28^. For instance, Riečanský and Katina^27^ suggested that the reduced alpha band power in the frontal cortical area during mental rotation task reflects enhanced attention allocation. These implications may be informative for understanding design neurocognition since engineering design seems highly visuospatial in nature^4,6,29^. Consequently, visuospatial thinking has been recognized as an essential skill in engineering design, necessary for generating and utilizing visual design representations^4,5,29^.

With the aim of contributing to the understanding of the neurocognition underlying generation and utilization of visual design representations, the presented study explored the effects of two types of projections in technical drawings (as 2D visual representations of technical systems) on engineers’ brain activity (measured using EEG) while CAD modelling based on them. Technical drawings visually present 3D objects (technical systems) in two dimensions (on the plane) using single- or multi-view projections^30^. Single-view projections represent the shape from an angle that provides information about all three principal dimensions while each dimension is equally distorted for a certain angle^5^. For example, in isometric projection the coordinate axes are distorted for 30° and spaced apart for 120°. On the other hand, multi-view projections contain several 2D views that provide design information, such as a set of three principal orthographic projections (front view, top view, left side view) in the first or third angle^30^. Hence, both types of projections describe the technical systems with the same contents and amount of information, but in different ways. Due to the distinction in the way of presenting design information, different neurocognitive responses to generating 3D CAD models from these projections were expected.

The initial analysis of the results (published in Lukačević et al.^31^) indicated higher power of brain activity when CAD modelling from orthographic than isometric projection. The differences in brain activity were revealed when the entire CAD task was considered^31^. However, generating 3D CAD models based on a technical drawing requires engineering skills to

1. interpret a technical system from a visual representation,
2. perceive a technical system in mind (generate its mental 3D model),
3. divide the visualized 3D mental representation of a technical system into features, and
4. externalize the 3D mental representation by generating its 3D visual representation in a CAD environment using Boolean primitives^6,32^.

Building on the initial work, the herein presented exploratory study further investigates differences in engineers’ brain activity while CAD modelling from orthographic and isometric projections by dividing the CAD task into two segments. The observed CAD task segments represent two of the listed engineering skills, which may be distinguished from the participants’ overt behaviour. In particular, CAD task segment #1 refers to interpreting a technical system from a visual representation (isometric or orthographic projection). In addition, CAD task segment #2 refers to generating a 3D CAD model based on the interpreted projections. Considering the division of the CAD task segments, the study aims to answer the following research questions:

*RQ1: Is the brain activity of mechanical engineers different when interpreting isometric (condition #1) and orthographic (condition #2) projections in technical drawings?*

*RQ2: Is the brain activity of mechanical engineers different when generating CAD models from isometric (condition #1) and orthographic (condition #2) projections in technical drawings?*

It is hypothesized that the mechanical engineers’ brain activity will be different when interpreting isometric and orthographic projections in technical drawings as well as when generating CAD models from them. The study analyses neural oscillations over the cortex, individual electrodes, cortical hemispheres, and cortical areas to answer research questions and test the hypothesis. The analysis focuses on theta (4–7 Hz), alpha (8–12 Hz), and beta (13–30 Hz) since previous EEG studies often reported changes in the power of these frequency bands when visually processing sets of visuospatial information—common in visuospatial tasks such as CAD modelling.

With reference to the results of the previous studies, an increase in theta and beta frequency bands and a decrease in alpha frequency band power (compared to the baseline) may be expected during both CAD task segments and for both conditions (#1 and #2). Additionally, larger theta and beta power increase and smaller alpha decrease may be expected when interpreting the orthographic projection and generating a 3D CAD model from it (compared to the condition with the isometric projection). Furthermore, it is assumed that the right hemisphere (RH) will be more activated than the left one as the RH in right-handed human beings seems specialised for processing visuospatial information^33^. For instance, Roberts and Ann Bell^34^ reported a larger alpha power decrease in the right than the left parietal area during visuospatial information processing, thus implying the importance of the RH and the frontal cortical area. Regarding the cortical areas, higher activation in frontal theta (increase), frontal beta (increase), and rear alpha (decrease) may be expected during both CAD task segments when using orthographic projections^26,35^. Such activation is often related to higher mental effort and cognitive workload, which is expected when using orthographic projection. Namely, it is assumed that one must allocate additional cognitive resources to mentally manipulate 2D information presented in the three 2D views of the orthographic projection to combine into a 3D mental model of the represented object^36^.

## Methods

### Study participants

The study recruited 20 male subjects to participate in the experiment. The inclusion criteria were: being a mechanical engineer, right-handed, and having at least a basic knowledge of using SolidWorks as CAD modelling software. In addition, the participants were instructed to refrain from coffee and caffeine beverages at least two hours before the experiment. Data of the two participants from the original sample (n = 20) were discarded from the data analysis. One participant reported diagnosed neurological issues, and the other left-handedness.

The participants filled in the questionnaire on demographics and prior-experiment experience as part of the experimental procedure. The results showed that participants ranged in age from 25 to 30, with a median (Med) of 27.50 and a median absolute deviation (MAD) of 1.34. Prior professional engineering experience of participants ranged from 0 to 72 months, with Med = 21.50 and MAD = 16.84. All the participants finished the same CAD course (as a part of their engineering studies) in which they learned how to use SolidWorks, a professional computer tool for 3D modelling and engineering documentation. They, on average, spend 10% of their work time on CAD modelling (Med = 10, MAD = 13.16), ranging from 0 to 70%. The distribution of their CAD modelling frequency was as follows: never (10%), rarely (20%), sometimes (25%), often (40%), and always (5%).

### Experimental tasks

**Figure 1:**
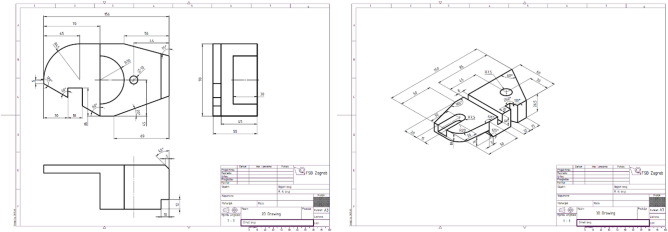
Orthographic projection of the part 1 (left) and isometric projection of the part 2 (right).

The study incorporated two CAD tasks in which the participants were asked to generate 3D CAD models of two parts based on their technical drawings. The complexity of the CAD tasks was kept the same, as defined by the type and the number of features constituting the resulting 3D models^37^. The parts consisted of the following features: a cuboid, a fillet, a chamfer, a through hole, a slot, and three through slots. The parts are presented in Fig. 1.

Participants were not restricted by the number or types of features when generating 3D models. In one CAD task, the technical system (part 1) was presented with the single-view isometric projection in the technical drawing (condition #1). In the other CAD task, the orthographic projection (condition #2) with three main views (front, top, right) in the first angle was used as a 2D visual representation (part 2). Hence, the projection type used to present the parts in technical drawings was an independent variable. In both cases, technical drawings were mediated by the monitor screen as the 2D interface.

### Experimental setup

**Figure 2:**
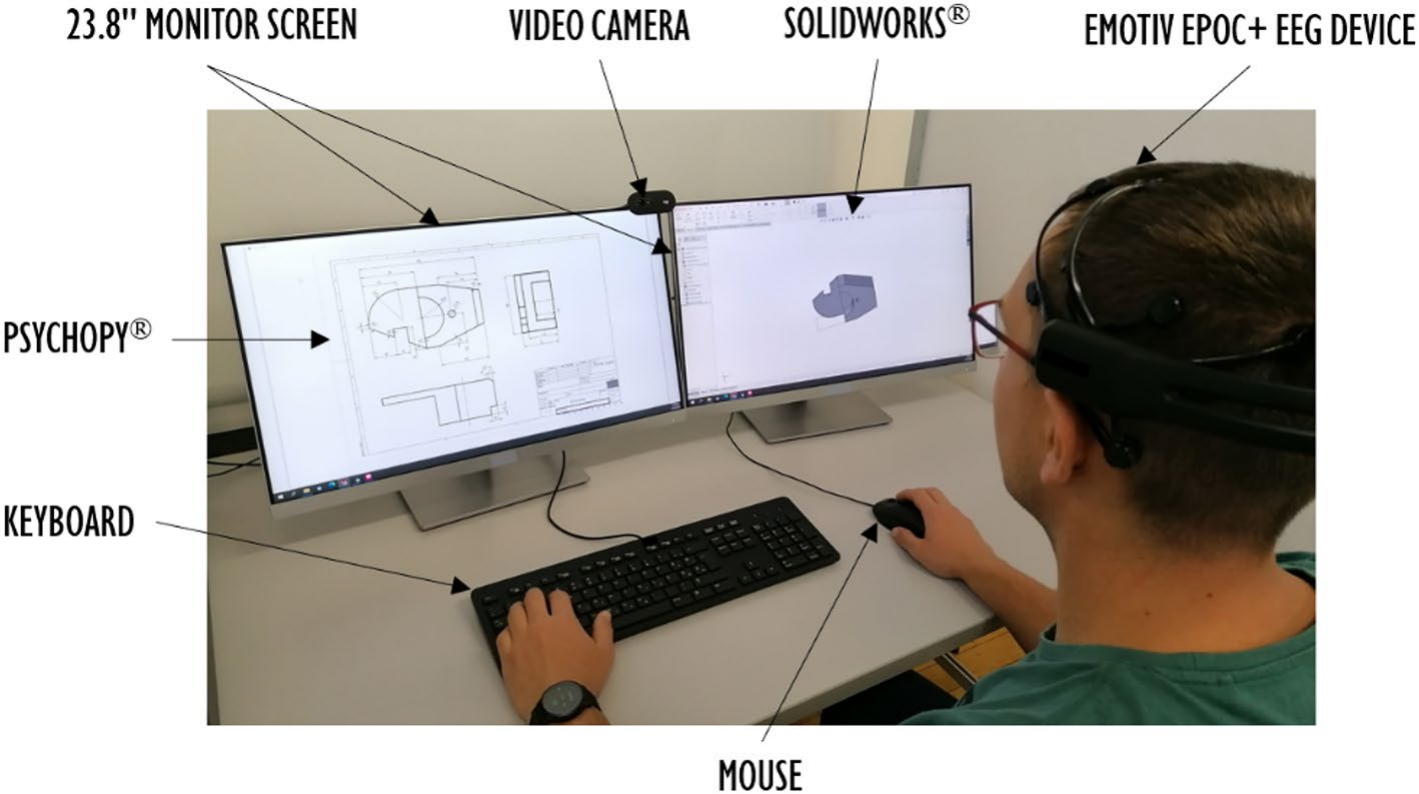
Experimental setup.

The experiment was conducted using one high-performance computer, two 23.8’’ monitor screens (resolution of 1920 × 1080 pixels, refresh rate of 60 Hz), and a keyboard and a mouse as the interaction devices. The technical drawings and instructions with detailed explanations of what should be done in each task and step were presented through the PsychoPy^38^ application on the left monitor. The CAD modelling was conducted in SolidWorks software, presented on the right monitor. As presented in Fig. 2, both screens were recorded for the entire experiment duration.

In addition, a video camera captured a participant’s face, but these data sets are not included in the herein-presented analysis. Screen and video recording enabled capturing behavioural data: experiment progress from the left screen and CAD modelling from the right screen. The PsychoPy enabled the synchronisation of behavioural and EEG data. The device used for EEG data gathering during the CAD tasks was Emotiv EPOC + with 14 electrodes (AF3, F7, F3, FC5, T7, P7, O1, O2, P8, T8, FC6, F4, F8, AF4) and the integrated amplifier. The sensors’ locations followed the international 10–20 system. Two reference sensors were at P3 and P4 locations. These locations were used as the reference in the later data pre-processing steps. Continuously captured EEG data was sent via wireless connection (Bluetooth Low Energy) to a high-performance computer. The sampling frequency used to collect EEG data was 128 Hz. According to the Nyquist-Shannon theorem, such sampling frequency is adequate for analysing frequency bands relevant to the study: theta (4–7 Hz), alpha (8–12 Hz), and beta (13–30 Hz)^39^.

### Experimental procedure

**Figure 3:**
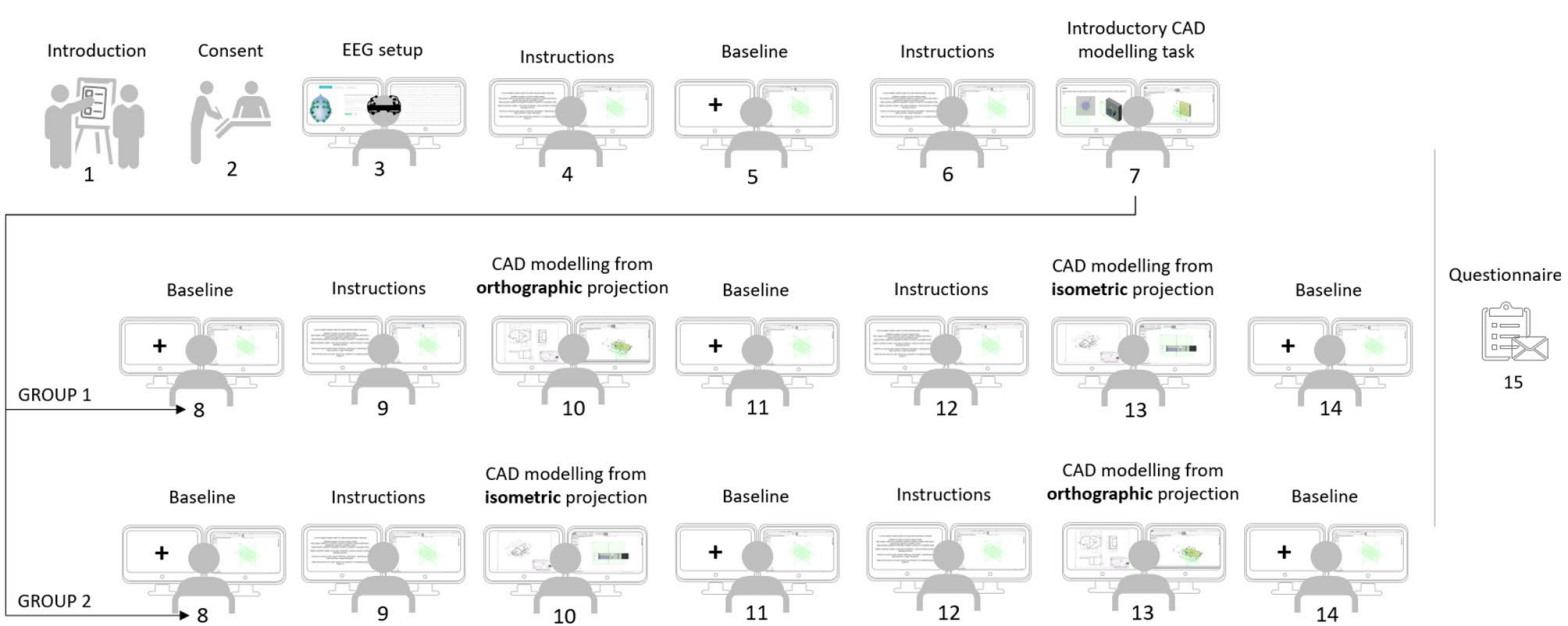
Experimental procedure.

The experimental procedure consisted of 15 steps, shown in Fig. 3. Firstly, the participants were introduced to the equipment and the experimental procedure. Next, participants were asked to sign a consent. The informed consent was obtained from all the participants. In the third step, the EEG headset was set up. The participants continued to the CAD tasks when the contact and EEG data quality were satisfactory (according to the metrics defined by Emotiv and indicated with green colour within the data gathering application).

The parts and the CAD tasks were the same for all the participants. The CAD tasks were not time limited. Each participant was asked to generate 3D CAD models of three parts in total. All the participants started with an introductory CAD task that served as a warm-up task for familiarisation with the interaction devices and CAD environment. The introductory CAD task consisted of seven actions: generation of three sketches and four features. The resulting 3D model contained a cuboid, a cylinder, a through hole, and chamfers. After the introductory CAD task, participants were instructed to generate 3D CAD models of two parts based on their drawings (as explained in the previous subsection). Each participant had one trial in each condition (orthographic or isometric projection). However, the order of the conditions was controlled; half of the participants (group 1) first generated a 3D CAD model of part 1 (from its isometric projection) and continued to part 2 (from its orthographic projection). The order was reversed for the other half of the participants (group 2). The randomized division was motivated by the goal of bypassing the potential bias of the previous task and cognitive fatigue as its consequence. Each CAD task was preceded and followed by a baseline task. For the baseline task, participants were asked to stare at the fixation cross on the monitor display until it disappeared (after 20 s). The slides with the instructions, the fixation cross, and technical drawings (presented on the left screen) advanced based on participants’ keyboard input. Thus, their timing was based on the duration of solving each step. The duration of the active part of the experiment (from step 4 to the end of step 14) ranged from 26.26 to 68.84 min, with Med = 45.07 min and MAD = 18.48 min. There were no breaks during the experiment. Finally, the questionnaire on demographics and prior-experiment experience related to CAD modelling and technical documentation was sent via e-mail to the participants after the experiment. The Ethics Committees of the Faculty of Mechanical Engineering and Naval Architecture, University of Zagreb approved the described experimental protocol. All the methods were carried out in accordance with relevant guidelines and regulations.

### Data pre-processing

**Figure 4:**
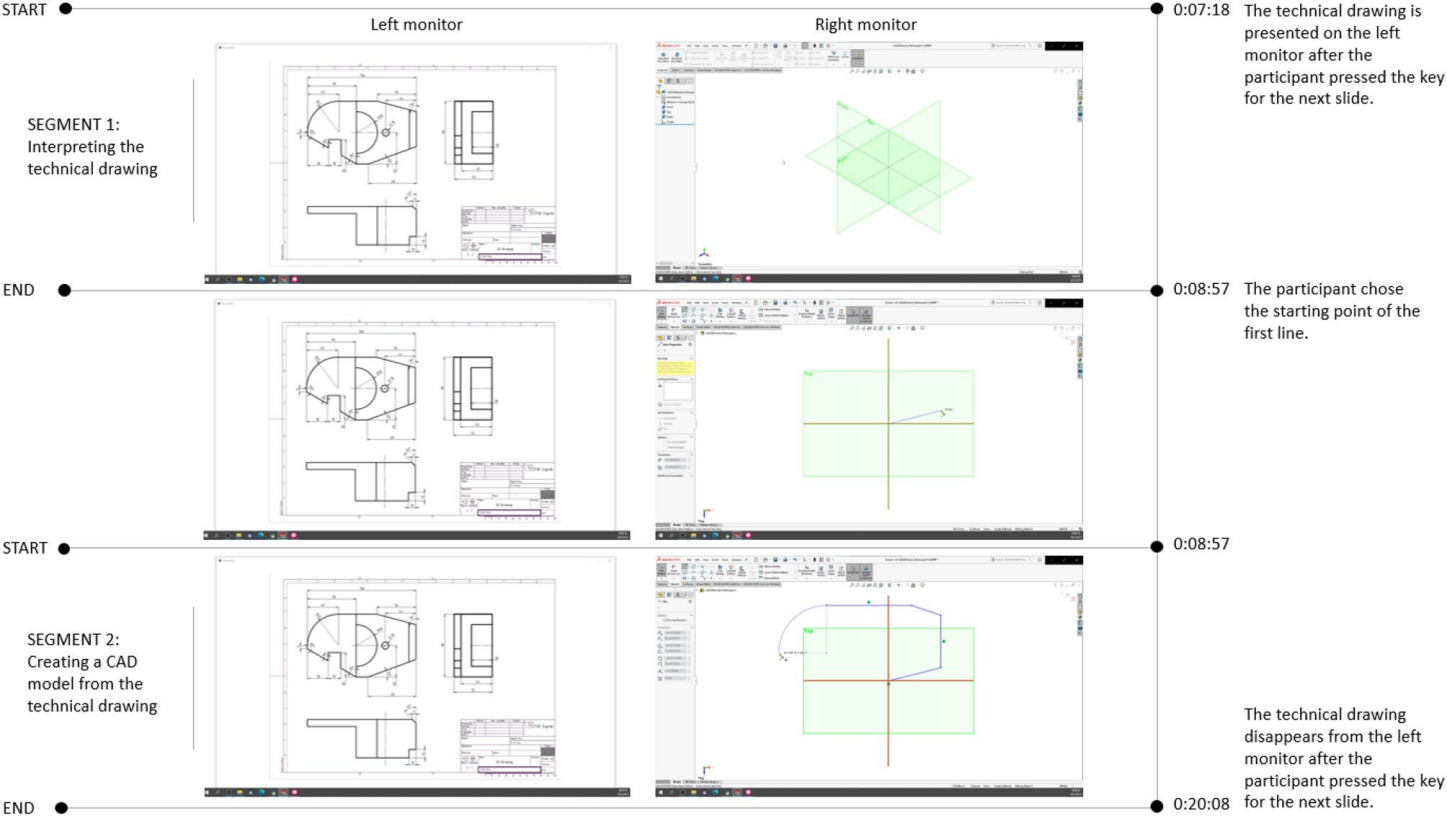
CAD task segmentation example.

The EEG data processing was conducted in MATLAB using the EEGLAB toolbox^40^. An original script for data processing was developed according to the pipelines described by Li et al.^41^, Vieira et al.^19^, and Jia et al.^42^. In the first step, DC offset specific for Emotiv EPOC + devices was removed with the infinite impulse response (IIR) filter (0.16 Hz first order high-pass filter). Secondly, frequencies outside the 4–45 Hz range were removed with the finite impulse response (FIR) filter. After that, muscle artefacts were removed with the blind source separation (BSS) technique based on canonical correlation analysis (CCA)^43^. The filtering parameters were set as follows: window length of 2.5 s, window shift of 1.2 s, and four as the number of the least correlated components to be removed. In the next step, the EEG recording was segmented into epochs representing the baselines and the tasks. Furthermore, the CAD tasks were segmented into CAD task segment #1—interpretation of a technical system from the 2D visual representation (isometric or orthographic) and CAD task segment #2—generation of a 3D CAD model. The start of the first CAD task segment was defined by the timing of the transition to the slide with 2D representation, and it was derived from the PsychoPy log files. The end of the first CAD task segment corresponds to the start of the second CAD task segment. Namely, the start of the CAD task segment #2 was defined as a moment when the participant started generating the first sketch element (e.g. by drawing the first line) in SolidWorks. An example of segmentation is presented in Fig. 4.

The segmentation was followed by removing of the windows (length of 1 s, shift of 1/128 s) with an amplitude exceeding $\pm$ 100 µV and/or the threshold value calculated for each participant individually. The threshold was calculated as a value three standard deviations greater than the mean (M) of the entire epoch across the electrodes. In this way, any 1 s long epoch of the EEG data with the M amplitude higher than the calculated threshold (or 100 µV if the absolute threshold value was above it) was discarded. The percentage of the bad EEG data ranged from 0.19 to 4.02% for the condition #1 (Med = 0.83, MAD = 0.76) and from 0 to 9.92% for the condition #2 (Med = 0.76, MAD = 0.94). In the next step, EEG data was divided into theta (4–7 Hz), alpha (8–12 Hz), and beta (13–30 Hz) sub-frequency bands using the FIR filter. After the threshold was applied, the power of EEG signals (Pow) was calculated as the M of the squared values, resulting from the band-pass filtering of the EEG signal and using Fast-Fourier Transformation. The EEGLAB function pop_eegfiltnew, hardcoded to Hamming window, was used for the filtering. In the final pre-processing step, task-related power (TRP) was calculated by subtracting the transformed Pow average of a subject *j* at an electrode *i* during a baseline task recorded before each CAD task from the transformed Pow average of a subject *j* at an electrode *i* during a CAD task. Hence, TRP values were calculated according to the following expression:

1 $${\mathrm{TRP}}_{ij}=\mathrm{log}{({\mathrm{Pow}}_{i}\left(\mathrm{task}\right)}_{j})-\mathrm{log}{({\mathrm{Pow}}_{i}\left(\mathrm{baseline}\right)}_{j}).$$

Positive TRP values reflect an increase of power during the CAD task (compared to the baseline task), whereas negative TRP values reflect a power decrease^44^.

### Data analysis

Data analysis was conducted using the R language. Descriptive statistics encompassed the calculation of the Med as a measure of central tendency and MAD as a measure of variability. These parameters were used for data distribution since they are robust to the effects of eventual outliers that potentially persisted after the data pre-processing. Besides, they are more suitable for describing the non-normal distributions (as tested by the Shapiro–Wilk test; p < 0.05). In addition, inferential tests enabled the calculation of differences in duration and TRP values between two CAD modelling task segments (interpreting a technical drawing and generating a 3D CAD model). The analysis encompassed a comparison of the tasks (their segments) based on TRP in three frequency bands (theta, alpha, and beta).

A nonparametric repeated measures ANOVA approach was adopted^45,46^ to study the differences in TRP values between the projections in each CAD task segment and in each frequency band. Such an approach is based on the Aligned Rank Transform (ART) procedure devised to handle data that violates ANOVA assumptions without inflating the Type I error rates^46^. For each setting (i.e. segment and frequency band), the factors of interest included the projection, electrode, hemisphere (LH and RH), and cortical area (frontal area -FA- and rear area -RA-). The odd-numbered electrodes were grouped under the LH, while the even-numbered ones were under the RH. To compare the TRP values between the cortical areas, the electrodes were distributed as follows: FA: AF3, F7, F3, F4, F8, AF4, FC5, FC6, and RA: O1, O2, P7, P8, T7, T8. The EEG device used in the experiment has good coverage of the FA, but a low spatial resolution in other areas (central, occipital, parietal, and temporal) since only two electrodes are in each of them. Consequently, the division into smaller cortical areas would offer results with a low statistical rigour.

Significant interaction and main effects detected using the nonparametric repeated measures ANOVA were further decomposed into simple interactions, simple main effects and pairwise comparisons to enable further insights. Herein, the pairwise Wilcoxon signed-rank test with Bonferroni correction was used for the posthoc comparisons. In addition to the (adjusted) p-values, the effect size (reported as r-value) of the Wilcoxon singed-rank test was calculated by dividing the test statistic by the square root of the number of observations. The p-values and the related effect size are, in the following section, coupled with the test statistic values; partial eta squared for the nonparametric factorial ANOVA test and V for the Wilcoxon signed-rank test. Significant differences are presented graphically in the box plots and numerically in the tables.

## Results

The first subsection presents the differences in duration when considering the entire CAD task and its two segments (CAD task segments #1 and #2). The second subsection compares the theta, alpha, and beta frequency band TRP values between conditions #1 and #2 for each CAD task segment. First, the significant differences in TRP values and/or large effect sizes are presented for the CAD task segment #1 in the following order. The significant main effects of projection (i.e. differences among projections considering the entire cortex) are discussed first. Then, the significant main effects and interactions concerning the cortical hemispheres are reported. Finally, the main effects and interactions concerning the cortical areas are listed. The same reporting structure is then followed for the CAD task segment #2. Note that, of the explored interactions, only several two-way interactions were found significant and are discussed accordingly (whereas insignificant results are omitted).

### Duration

**Figure 5:**
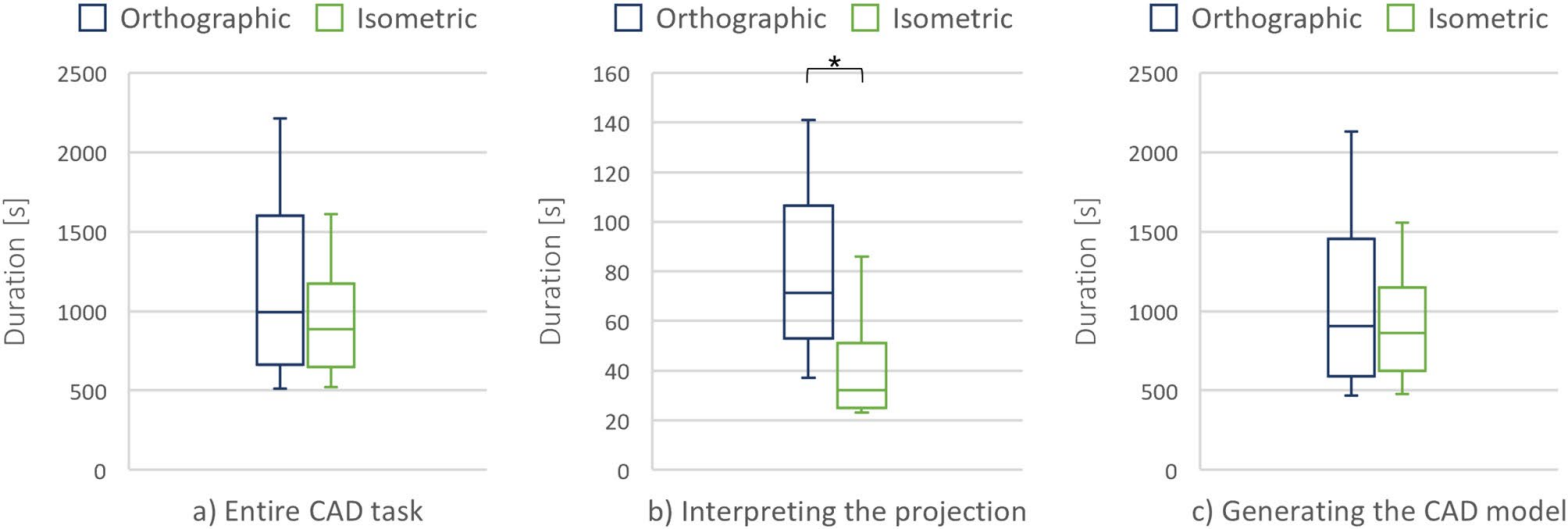
Duration of the (**a**) entire CAD task, (**b**) first CAD task segment, and (**c**) second CAD task segment.

The average duration of the CAD task performance (expressed in seconds) was higher when using the orthographic (Med = 991.04, MAD = 405.29) than the isometric projection (Med = 886.44, MAD = 360.26), as shown in Fig. 5a. However, the difference between the two conditions was not statistically significant.

The completion time of the first CAD task segment (interpreting the 2D visual representations) was significantly longer when interpreting the orthographic (Med = 71.5, MAD = 37.81) than the isometric (Med = 32, MAD = 12.6) projection, with V = 170, p = 1.53·10^–5^, and r = 0.87.

Participants spent similar time generating CAD models from the orthographic (Med = 904, MAD = 473.69) and the isometric (Med = 861, MAD = 360.27) projections. Consequently, the completion time of the second task segment was slightly, but not significantly different.

### Theta, alpha, and beta TRP

#### CAD task segment 1: interpreting the projections

##### Cortex (considering all the 14 electrodes)

**Figure 6:**
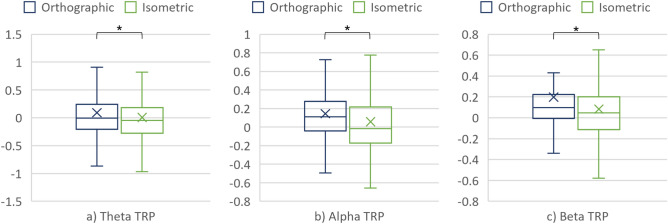
(**a**) Theta TRP, (**b**) Alpha TRP, and (**c**) Beta TRP over the cortex when interpreting the projections.

The nonparametric repeated measures ANOVA revealed a significant main effect of the projection on the TRPs in all three frequency bands. For completeness, the differences in theta, alpha, and beta TRPs over the cortex when interpreting the orthographic and isometric projection were also assessed using the Wilcoxon signed rank test. Figure 6 and Table 1 give further details on these differences.

**Table 1:** Comparison of the TRPs over the cortex when interpreting the projections.

Frequency band	Projection	Med	MAD	ART ANOVA		Wilcoxon	
				F, p	ηp2	V, p	r
Theta TRP	Orthographic	− 8.32×10^–3^	0.33	5.61, 1.82×10^–2^	1.21×10^–2^	13,426, 3×10^–2^	0.14
	Isometric	− 4.61×10^–2^	0.32				
Alpha TRP	Orthographic	0.11	0.24	22.09, 3.44×10^–6^	4.59×10^–2^	12,188, 1.2×10^–3^	0.2
	Isometric	− 1.81×10^–2^	0.27				
Beta TRP	Orthographic	9.65×10^–2^	0.16	11.81, 6.44×10^–4^	2.51×10^–2^	12,864, 7.94×10^–3^	0.17
	Isometric	4.88×10^–2^	0.23				

##### Cortical hemispheres

The main effect of the cortical hemisphere was found on the theta and alpha TRPs (consult Table 2 for details).

**Table 2:** The main effect of the cortical hemisphere.

Frequency band	Cortical hemisphere	Med	MAD	ART ANOVA		Wilcoxon	
				F, p	ηp2	V, p	r
Theta TRP	LH	5.98×10^–2^	0.31	47.68, 1.68×10^–11^	9.41×10^–2^	23,412, 1.68×10^–11^	0.41
	RH	− 0.13	0.31				
Alpha TRP	LH	6.99×10^–2^	0.25	6.96, 8.61×10^–3^	1.49×10^–2^	19,673, 1.27×10^–3^	0.2
	RH	3.91×10^–2^	0.28				

**Figure 7:**
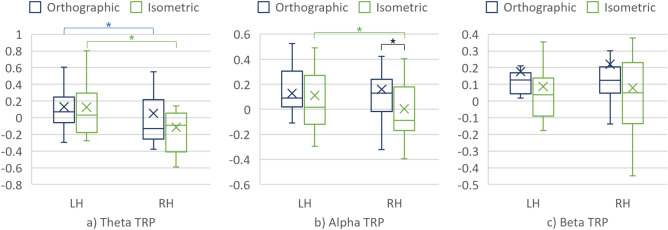
(**a**) Theta TRP, (**b**) Alpha TRP, and (**c**) Beta TRP over the cortical hemispheres when interpreting the projections.

No significant interaction effects between hemisphere and the remaining factors were found in the three frequency bands. Nevertheless, pairwise Wilcoxon signed rank tests with Bonferroni correction revealed a significant difference in alpha TRP between the projections over the RH. In addition, alpha TRP significantly differed between the hemispheres, but only for the condition with the isometric projection, whereas significant differences in theta TRP were found between hemispheres when interpreting both projections (Fig. 7 and Table 3).

**Table 3:** Significant differences in TRP regarding the cortical hemispheres and projections.

Frequency band	Projection	Cortical hemisphere	Med	MAD	Wilcoxon	
					V, p	r
Theta TRP	Orthographic	LH	6.49×10^–2^	0.27	6373, 7.68×10^–9^	0.3
		RH	− 0.12	0.28		
	Isometric	LH	4.37×10^–2^	0.34	5404, 6.36×10^–4^	0.52
		RH	− 0.17	0.35		
Alpha TRP	Isometric	LH	2.22×10^–2^	0.25	5713, 3.07×10^–5^	0.37
		RH	− 7.02×10^–2^	0.27		
Alpha TRP	Orthographic	RH	0.12	0.24	2964, 1.2×10^–2^	0.26
	Isometric		− 7.02×10^–2^	0.27		

##### Cortical areas

**Figure 8:**
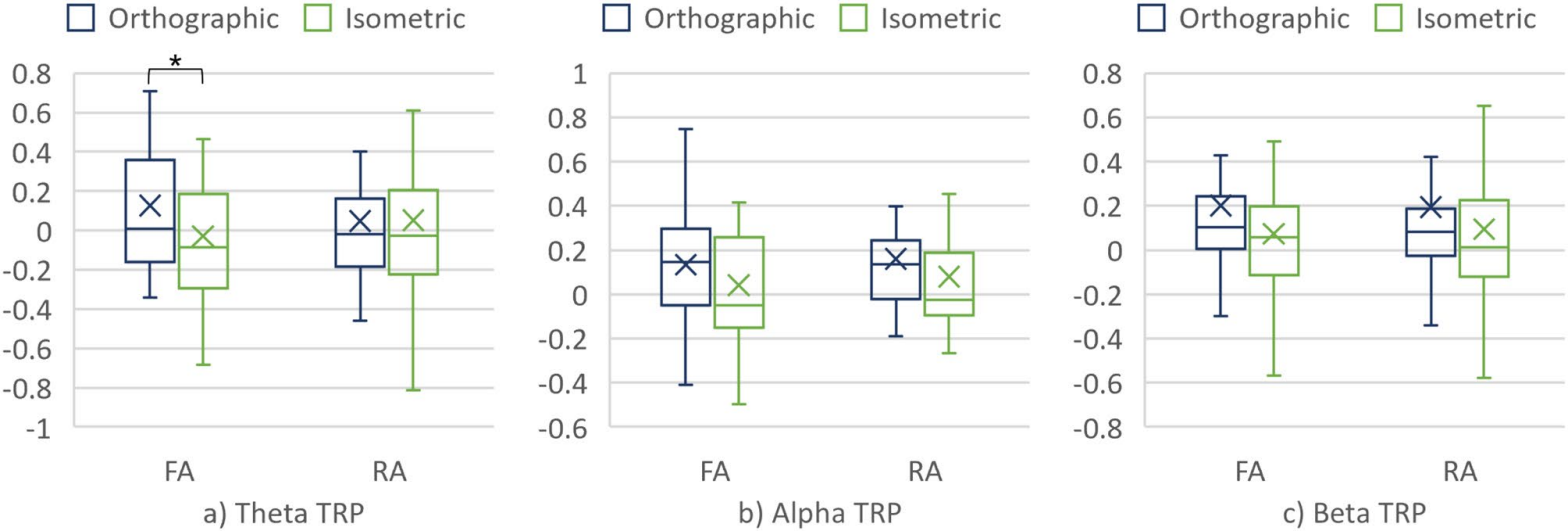
(**a**) Theta TRP, (**b**) Alpha TRP, and (**c**) Beta TRP over the cortical areas when interpreting the projections.

No main effects of the cortical area were found in the TRP values of theta, alpha, and beta when interpreting the projections. However, there was a statistically significant interaction between the projection and the cortical area in the theta frequency band (F = 7.56, p = 6.22·10^–3^, and ηp^2^ = 1.62·10^–2^). As shown in Fig. 8a, further analysis of the simple main effects of projection showed significant differences between the theta TRP over the FA, with V = 3603, p = 1·10^–3^, and r = 0.13.

### CAD task segment 2: generating the CAD models

#### Entire cortex (considering all the 14 electrodes)

**Figure 9:**
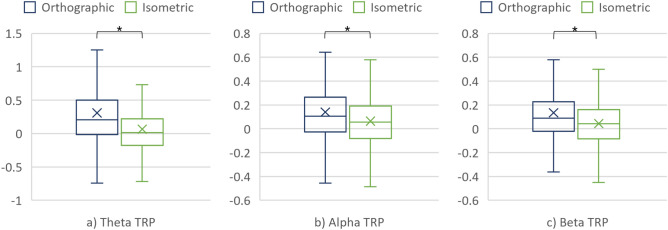
(**a**) Theta TRP, (**b**) Alpha TRP, and (**c**) Beta TRP over the cortex when generating the 3D CAD models.

The main effect of the projection on the TRPs over the cortex was found in all three frequency bands when generating the CAD models. As presented in Fig. 9 and Table 4, theta, alpha, and beta TRPs over the entire cortex significantly different when interpreting the orthographic and isometric projections.

**Table 4:** Comparison of the TRPs over the cortex when interpreting the projections.

Frequency band	Projection	Med	MAD	ART ANOVA		Wilcoxon	
				F, p	ηp^2^	V, p	r
Theta TRP	Orthographic	0.2	0.37	61.4, 3.27×10^–14^	0.12	13,426, 3×10^–2^	0.14
	Isometric	1.2×10^–2^	0.29				
Alpha TRP	Orthographic	0.11	0.22	9.74, 1.92×10^–3^	2.08×10^–2^	12,188, 1.2×10^–3^	0.20
	Isometric	5.62×10^–2^	0.2				
Beta TRP	Orthographic	9.01×10^–2^	0.18	16.73, 5.1×10^–5^	3.52×10^–2^	12,864, 7.94×10^–3^	0.17
	Isometric	4.16×10^–2^	0.18				

#### Cortical hemispheres

The main effect of the cortical hemisphere was found on the TRPs in all three frequency bands (see Table 5).

**Table 5:** The main effect of the cortical hemisphere.

Frequency band	Hemisphere	Med	MAD	ART ANOVA		Wilcoxon	
				F, p	ηp2	V, p	r
Theta TRP	LH	0.16	0.36	29.45, 9.31×10^–8^	6.03×10^–2^	22,141, 8.59×10^–8^	0.34
	RH	3.58×10^–2^	0.31				
Alpha TRP	LH	0.12	0.21	10.92, 1.03×10^–3^	2.32×10^–2^	20,656, 4.66×10^–4^	0.26
	RH	3.85×10^–2^	0.22				
Beta TRP	LH	8.19×10^–2^	0.2	6.49, 1.12×10^–2^	3.89×10^–3^	20,420, 1.1×10^–4^	0.24
	RH	4.46×10^–2^	0.17				

**Figure 10:**
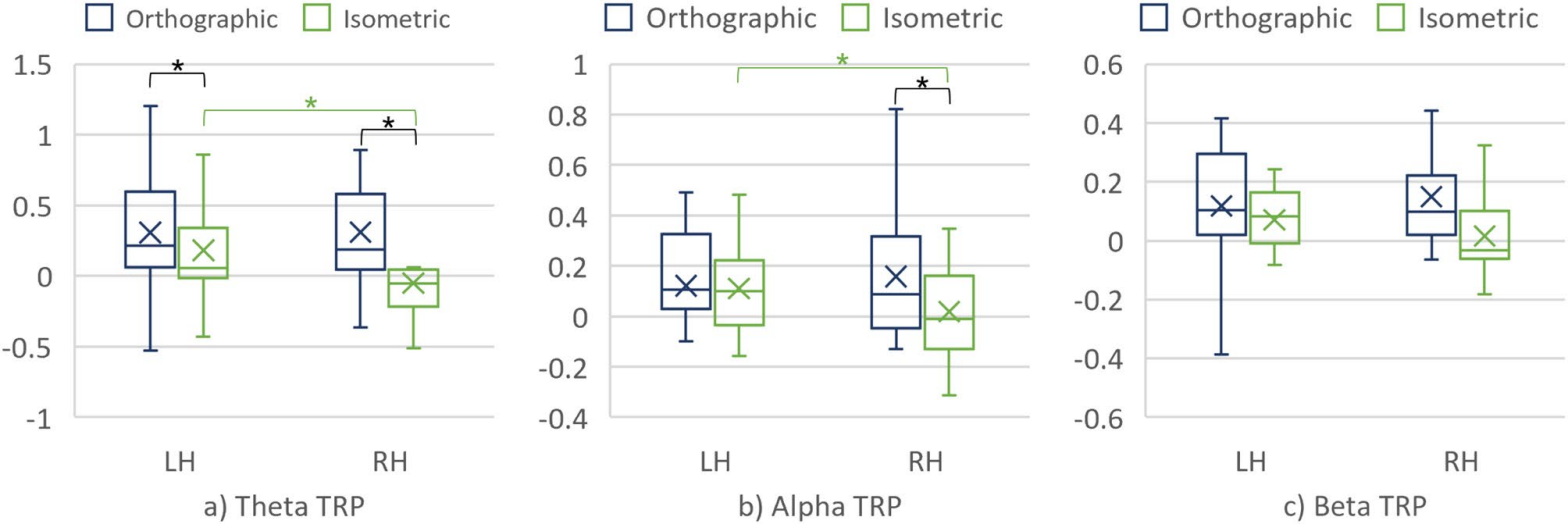
(**a**) Theta TRP, (**b**) Alpha TRP, and (**c**) Beta TRP over the cortical hemispheres when generating the 3D CAD models.

Furthermore, the interaction effect of the projection and cortical hemisphere was found in theta (F = 5.14, p = 2.38×10^–2^, and ηp^2^ = 1.11·10^–2^) and alpha (F = 3.73, p = 0.05, and ηp^2^ = 8.05·10^–3^) frequency bands. Decomposing the two-way interaction into simple main effects revealed the significant differences between the projections in the theta TRPs over both hemispheres. In other words, the simple main effect of the projection was significant both in the LH (V = 2492, p = 2.41·10^–4^, r = 0.33) and the RH (V = 1414, p = 3.05·10^–10^, r = 0.56), as presented in Fig. 10a. In addition, simple main effect of the cortical hemisphere was significant in the condition with the isometric projection (V = 6511, p = 9.88·10^–2^, and r = 0.55).

At the electrode level, difference between the LH and the RH was found in all electrodes for the isometric projection condition. These electrodes are presented in Table 6.

**Table 6:** Difference between the LH and the RH in theta TRP over the individual electrodes.

Projection	Electrode	LH		RH		LH vs RH	
		Med	MAD	Med	MAD	V, p	r
Isometric	AF3|AF4	0.17	0.24	− 7.16×10^–2^	0.29	165, 1.07×10^–4^	0.43
	F3|F4	4.71×10^–2^	0.29	− 7.8×10^–2^	0.13	169, 2.29×10^–5^	0.21
	F7|F8	3.84×10^–2^	0.24	− 0.13	0.22	154, 2×10^–3^	0.38
	FC5|FC6	0.14	0.4	− 0.11	0.29	168, 3.81×10^–5^	0.42
	T7|T8	0.21	0.38	− 9.98×10^–2^	0.33	152, 2×10^–3^	0.37
	P7|P8	5.65×10^–2^	0.43	− 4.04×10^–2^	0.32	153, 2×10^–3^	0.22
	O1|O2	0.15	0.34	1.44×10^–2^	0.11	132, 4.3×10^–2^	9.49×10^–2^

In addition, the interaction effect of the projection and cortical hemisphere area was found for alpha frequency band. Decomposing this interaction into simple main effect of the projection revealed the differences in alpha TRP over the RH, as presented in Fig. 10b, with V = 2964, p = 1.2·10^–2^, r = 0.23. Furthermore, the simple main effect of the cortical hemisphere was found on alpha TRP for the condition with the isometric projection, with V = 5713, p = 3.07·10^–5^, r = 0.37. Finally, significant differences in alpha TRP between the LH and the RH were found at several electrodes presented in Table 7.

**Table 7:** Difference between the LH and the RH in alpha TRP over the individual electrodes.

Projection	Electrodes	LH		RH		LH vs RH	
		Med	MAD	Med	MAD	V, p	r
Isometric	AF3|AF4	0.13	0.23	3.78×10^–2^	0.22	139, 1,8×10^–2^	0.2
	F3|F4	0.12	0.14	4.84×10^–2^	0.17	132, 4.3×10^–2^	8.44×10^–2^
	F7|F8	0.12	0.24	− 2.65×10^–2^	8.97×10^–2^	135, 3×10^–2^	0.25
	T7|T8	0.14	0.2	1.8×10^–2^	0.27	133, 3.8×10^–2^	0.23
Orthographic	T7|T8	9.16e−2	0.14	5.47×10^–2^	0.16	139, 1.8×10^–2^	0.18

##### Cortical area

**Figure 11:**
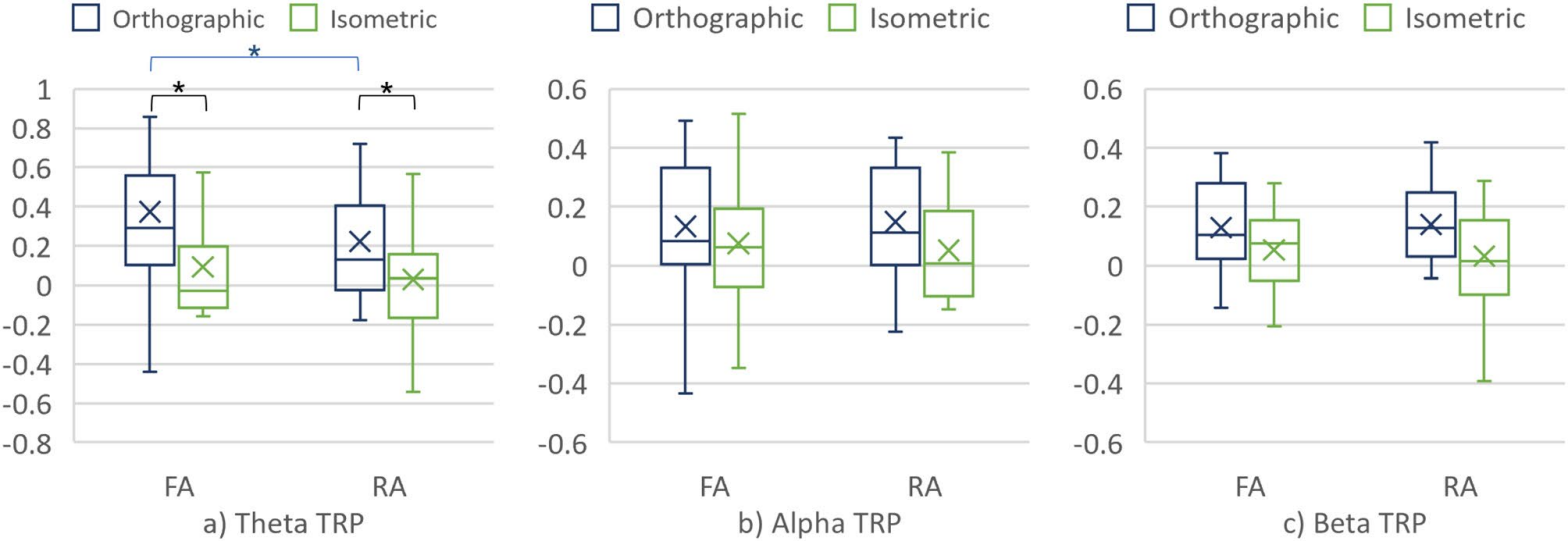
(**a**) Theta TRP, (**b**) Alpha TRP, and (**c**) Beta TRP over the cortical areas when generating the 3D CAD models.

The main effect of the cortical area was found on theta TRP, with F = 13.88, p = 2.19·10^–4^, and ηp^2^ = 2.94·10^–2^. Furthermore, the interaction effect of the projection and cortical area was found in the same (theta) frequency band (F = 9.99, p = 1.67·10^–3^, and ηp^2^ = 2.13·10^–2^). Further decomposing the interaction into the simple main effects revealed significant differences in theta TRP over both the FA and the RA when compared between the projections (see Fig. 11 and Table 8). In addition, the simple main effect of the cortical area was found on theta TRP when generating the CAD model from the orthographic projection.

**Table 8:** Simple main effects on theta TRP.

Cortical area	Projection	Med	MAD	Wilcoxon	
				V, p	r
FA	Orthographic	0.31	0.42	1921, 4.76×10^–11^	0.33
	Isometric	7.25×10^–3^	0.29		
RA	Orthographic	9.88×10^–2^	0.3	1937, 2×10^–3^	0.17
	Isometric	3.47×10^–2^	0.31		
FA	Orthographic	0.31	0.42	9608, 1×10^–3^	0.2
RA		9.88×10^–2^	0.3		

##### Individual electrodes

**Figure 12:**
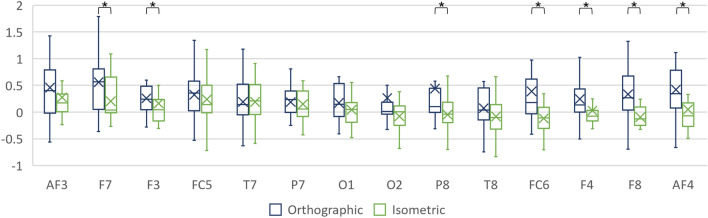
Theta TRP at the individual electrodes when generating the CAD models.

For completeness, the interaction among projection and individual electrodes (encoded as: AF3, AF4, F3, F4, F7, F8, FC5, FC6, P7, P8, T7, T8, O1 and O2) was studied. No significant interaction between projection and individual electrodes factors was found in any of the bands. Nevertheless, pairwise Wilcoxon signed rank tests with Bonferroni correction identified several electrodes at which theta TRP differs significantly between the projections (Fig. 12 and Table 9).

**Table 9:** Theta TRP at and among the individual electrodes.

Electrode	Orthographic		Isometric		Orthographic vs isometric	
	Med	MAD	Med	MAD	V, p	r
F7	0.56	0.56	3.84×10^–2^	0.24	37, 3.4×10^–2^	0.24
F3	0.24	0.3	4.71×10^–2^	0.29	19, 2×10^–8^	0.26
P8	0.1	0.23	− 4.04×10^–2^	0.32	35, 2.7×10^–2^	0.31
FC6	0.18	0.42	− 0.11	0.29	21, 3×10^–3^	0.66
F4	0.13	0.26	− 7.8×10^–2^	0.13	28, 1×10^–2^	0.42
F8	0.27	0.34	− 0.13	0.22	16, 1×10^–3^	0.52
AF4	0.34	0.43	− 7.16×10^–2^	0.29	21, 3×10^–8^	0.46

## Discussion

Engineers’ brain activity was captured and analysed during the visuospatially-intensive design tasks of CAD modelling in two conditions—when technical systems were presented with isometric (condition #1) and orthographic (condition #2) projections in technical drawings. An increase in theta and beta TRP (compared to the baseline) was expected in both CAD task segments (interpreting the projections in technical drawings as segment #1 and generating the 3D CAD models from them as segment #2) since it has often been related to cognitive processing of visuospatial information^22–25^. In line with that assumption, beta TRP increased in both CAD task segments. However, theta TRP increased when generating the 3D CAD models while surprisingly decreased when interpreting the technical drawings (for both conditions). In addition, alpha TRP decreased only when interpreting the isometric projection. A decrease of alpha TRP was expected in both CAD task segments and for both conditions since such a response has often been observed when processing visuospatial information^27,28^. Moreover, it is generally considered that the RH is specialised for processing visuospatial information in right-handed human beings^33^. Since the tasks used in the experiment are visuospatially-intensive and all the participants included in the analysis were right-handed, higher activation over the RH was expected when performing the CAD tasks. However, the TRP values over the RH were either similar to those of the LH (theta TRP during the first CAD task segment) or lower (in all the other cases).

A possible explanation for unexpected brain activity may be related to cognitive characteristics of the CAD tasks and visuospatial information processing in the CAD context. In addition to that argument, Willis et al.^47^ suggested that brain activity in visuospatial information processing highly depends on the task requirements posed to the participants (what they should do with presented information) and not only the type of information that should be processed. For example, several studies have reported increased alpha TRP while solving standardized tests related to aspects of visuospatial thinking^23,25,33^. Hence, it may be that increase in alpha TRP revealed when performing CAD tasks is due to using similar cognitive mechanisms. Similarly, revealed brain activity in individual hemispheres aligns with the results reported by Ornstein et al.^33^ and Roberts and Ann Bell^34^, who argued that higher activation of the LH may be caused by the analytic strategies specific for the mental rotation tasks. Both mental rotation and interpretation of the visual representation ask for a visual transformation as an aspect of visuospatial thinking (according to Shah et al.^29^). Hence, it may be that interpretation of visual representations causes higher activation of the LH because of the underlying visual transformation required to conduct it. Furthermore, the dimensionality of visual representation used in the mental rotation task seems to affect alpha TRP in the hemispheres. Namely, the 2D mental rotation task was previously associated with the higher parietal activation in the LH than the RH. On the contrary, the activation was higher in the right than the left parietal area during the 3D mental rotation task^34^. It is yet to be explored why the results diverge across the studies that used the same standardized tests and what it means for the CAD context.

The study hypothesised differences in mechanical engineers’ brain activity when interpreting isometric and orthographic projections in technical drawings as well as when generating CAD models from them. EEG results revealed statistical significance and considerable effect sizes in both CAD task segments when analysed across the cortex, cortical hemispheres, cortical areas, and individual electrodes. Thus, both research questions are answered positively, and the hypothesis is confirmed; the brain activity of mechanical engineers was different when interpreting isometric and orthographic projections in technical drawings, as well as when generating 3D CAD models from them.

Moreover, the analysis revealed a significant difference in the duration of the first CAD task segment (interpretation of the technical drawing) between the orthographic and isometric projection conditions. In contrast, the time spent generating the 3D geometry with the CAD tool (as the second CAD task segment) was similar in both conditions. Hence, the duration differences imply a significantly faster visual transformation and synthesis^29^ (prevalent in the first CAD task segment) from the isometric than the orthographic projection. Considering these differences in the duration of performing the CAD task segments, a more evident effect of the projections on engineers’ brain activity was expected when comparing brain activity in CAD task segment #1.

However, the following subsections imply different conclusions. In particular, it seems that the effect of the projections on engineers’ brain activity is more evident when generating CAD models than interpreting the technical drawings. Such results may be related to different aspects underlying the two CAD task segments (visual transformation and synthesis for segment #1; visual expression for segment #2). Differences in brain activity between the CAD task segments will be analysed in future work.

### Differences in mechanical engineers’ brain activity when interpreting the projections

The analysis of the TRP values in all three frequency bands suggested statistically significant differences in brain activity when interpreting the orthographic and isometric projections. In particular, a decrease of theta TRP was smaller when interpreting the orthographic projection (as compared to the baseline). Furthermore, alpha TRP increased when interpreting the orthographic projection while decreased when using the isometric one. Finally, an increase in beta TRP was larger for the orthographic than the isometric projection. The smaller decrease or larger increase in theta and beta TRP confirm the assumption of using more cognitive resources to interpret the technical drawing in which the technical system is presented with the orthographic projection (due to the need to combine three 2D views to a mental 3D model). Such results are in line with previous studies (e.g. the work of Fajen and Philips^36^). Alpha, on the other hand, behaved differently from what was expected—there was more alpha TRP in condition #2 although it was expected to see alpha TRP decrease with increasing processing demands (imposed by the orthographic projection)^27,28^.

Moreover, mechanical engineers used the cortical hemispheres similarly when interpreting both projections since no significant differences in the TRP values were found between the hemispheres when considering the projections. However, alpha TRP suggests different activation of the RH when interpreting the isometric than the orthographic projection. In addition, a significant difference in alpha TRP was found between the LH and the RH when interpreting the projections. Similarly, significant differences were found in theta frequency band when TRPs were compared between the hemispheres. These results imply the asymmetric hemisphere activation when interpreting the projections.

### Differences in mechanical engineers’ brain activity when generating the 3D CAD models from the projections

Similarly to the first CAD task segment, the TRP values were significantly different when generating the CAD models from the orthographic and isometric projections in all three frequency bands. However, the difference in TRP values for the CAD task segment #2 between the conditions was reflected in a larger increase when using the orthographic than the isometric projection in all three frequency bands.

Furthermore, significant differences in theta TRP were found over the individual electrodes when comparing the conditions (see Fig. 12). These electrodes were mainly located in the FA, thus implying the important role of theta over the FA in distinguishing the effects of the projections on engineers’ brain activity.

The effect of the projections when generating the CAD models was reflected in the theta and alpha TRPs, which both significantly differed for conditions #1 and #2 over the RH. The difference among the projections in theta TRP was also significant over the LH. When comparing theta TRP values over the hemispheres, it is noticeable that a decrease (with respect to the baseline task) is present over the RH and only for the isometric condition. In addition, the differences in TRP values over the LH and the RH were significant in the theta and alpha frequency band when generating the CAD models from the isometric projection. For this condition (#1), the revealed difference in theta TRP between the hemispheres was corroborated by the significant differences among all the seven pairs of the electrodes (as shown in Table 6). At the level of the electrodes, the effect of the hemispheres on the alpha TRP was noticed mainly over those located in the FA (see Table 7).

Considering the cortical areas, significant differences between the conditions were found in theta TRP over both the RA and the FA. Theta TRP over the FA increases in value for the orthographic and a decreased for the isometric projection. Such results are aligned with previous studies on the visual processing of information. For example, Liu et al.^26^ reported high theta TRP over the FA during a mental rotations task. An increase in theta TRP over the FA for condition #2 might be related to higher requirements on attention and the level of the cognitive load imposed by interpreting and generating CAD models from the orthographic projection^28^. Higher alpha TRP in the RA for condition #2 in both CAD task segments suggests similar explanations (although differences were not statistically significant). For instance, Gerlic and Jausovec^35^ related higher FA alpha power with more efficient processing of presented information and higher alpha activation in the RA (temporal area in particular) with higher cognitive load.

### Limitations

Several limitations of the presented study should be noted. The first limitation concerns the sample size. Namely, the statistical analysis was conducted on data from the 18 participants and offered some statistical evidence. However, the study should be repeated with more participants to corroborate the recognised differences statistically. In addition, both participants whose data were discarded from the analysis belonged to group two (as defined in Fig. 3), which started CAD modelling from the isometric and then moved to the orthographic projection. A difference in the number of participants within the groups could have affected the lack of statistical significance. Furthermore, the study should be more extensive with the capabilities of the used EEG device. Namely, the EEG device used in the study contained 14 electrodes. Because of the relatively low spatial resolution, the study did not try to relate brain activity and functions of brain areas where the electrodes were located since such analysis may not be reliable^48^.

## Conclusion and further work

The presented study showed that engineers’ brain activity in interpreting the 2D visual representations of technical systems (technical drawings with orthographic and isometric projections) and CAD modelling from them can be recorded, described, and compared using EEG. The results imply the sensitivity of engineers’ brain activity in CAD modelling to the visual representation from which a technical system is interpreted. In particular, when interpreting the technical drawings and CAD modelling from them, significant differences were found in theta, alpha, and beta TRP over the cortex (considering all 14 electrodes cumulatively). Furthermore, the results revealed significant differences in theta and alpha TRP when considering the individual electrodes, the cortical hemispheres, and the cortical areas. In particular, theta TRP over the RH and the FA seems to be essential in distinguishing neurocognitive responses to the orthographic and isometric projections. In further analysis, we will aim to relate brain activity with CAD actions, shorter task epochs, and outcomes. The conducted exploratory study sets the foundations for exploring engineers’ brain activity while solving visuospatially-intensive design tasks, whose segments are relatable to the aspects of visuospatial thinking. Future work will explore brain activity in other design activities that are highly visuospatial, with a larger sample size and the EEG device of a higher spatial resolution. As a result, typical brain behaviour(s) for visuospatially-intensive design activities may be understood, described, and used as input when further developing visual representations of technical systems, CAD software, and HCI tools.

## Data Availability

The datasets generated and analysed during the current study are available in the Zenodo repository at the following link https://doi.org/10.5281/zenodo.7185167.

## References

[1] Ullman DG, Wood S, Craig D (1990). The importance of drawing in the mechanical design process. Comput. Graph..

[2] Hubka, V. *Principles of engineering design*. vol. 46 (Butterworth Scientific, 1980).

[3] Pei E, Campbell I, Evans M (2011). A taxonomic classification of visual design representations used by industrial designers and engineering designers. Des. J..

[4] Tovey M (1986). Thinking styles and modelling systems. Des. Stud..

[5] Lieu, D. K. & Sorby, S. *Visualization, modeling, and graphics for engineering design*. vol. 148 (Cengage Learning, 2016).

[6] Ullman DG (2010). The Mechanical Design Process. McGraw-Hill.

[7] Summers JD, Shah JJ (2004). Representation in engineering design: A framework for classification. Proc. ASME Des. Eng. Tech. Conf..

[8] Goldschmidt G (2007). To see eye to eye: The role of visual representations in building shared mental models in design teams. CoDesign.

[9] Boa, D. & Hicks, B. Information operations: A model for characterising information interaction of engineers. in *Analyzing Cognitive Processes during Design: Proceedings of the HBiD 2014* (eds. Meboldt, M. & Matthiesen, S.) (2014).

[10] Norman, K. L. Spatial visualization—a gateway to computer-based technology. *J. Spec. Educ. Technol.* **XII**, (1994).

[11] Goel AK, Vattam S, Wiltgen B, Helms M (2012). Cognitive, collaborative, conceptual and creative—Four characteristics of the next generation of knowledge-based CAD systems: A study in biologically inspired design. CAD Comput. Aided Des..

[12] Goldschmidt G (1994). On visual design thinking: The vis kids of architecture. Des. Stud..

[13] Suwa M, Tversky B (1997). What do architects and students perceive in their design sketches? A protocol analysis. Des. Stud..

[14] Gero J, Milovanovic J (2020). A framework for studying design thinking through measuring designers’ minds, bodies and brains. Des. Sci..

[15] Hay L, Cash P, McKilligan S (2020). The future of design cognition analysis. Des. Sci..

[16] Vieira S, Benedek M, Gero J, Li S, Cascini G (2022). Design spaces and EEG frequency band power in constrained and open design. Int. J. Des. Creat. Innov..

[17] Jia W, Zeng Y (2021). EEG signals respond differently to idea generation, idea evolution and evaluation in a loosely controlled creativity experiment. Sci. Rep..

[18] Vieira S (2020). The neurophysiological activations of novice and experienced professionals when designing and problem-solving. Proc. Des. Soc. Des. Conf..

[19] Vieira S (2020). The neurophysiological activations of mechanical engineers and industrial designers while designing and problem-solving. Des. Sci..

[20] Borgianni Y, Maccioni L (2020). Review of the use of neurophysiological and biometric measures in experimental design research. Artif. Intell. Eng. Des. Anal. Manuf..

[21] Nguyen TA, Zeng Y (2010). Analysis of design activities using EEG signals. Int. Des. Eng. Tech. Conf. Comput. Inf. Eng. Conf..

[22] Antonenko P, Paas F, Grabner R, van Gog T (2010). Using electroencephalography to measure cognitive load. Educ. Psychol. Rev..

[23] Promsorn, P., Boonyahotra, V. & Sittiprapaporn, P. Spatial Abilities Improve Brain-Computer Interface Performance Indexed by Electroencephalography. in *14th International Conference on Electrical Engineering/Electronics, Computer, Telecomunications and Information Technology* 34–37 (2017).

[24] Nguyen, T. A. & Zeng, Y. Clustering designers’ mental activities based on eeg power. *Tools Methods Compet. Eng.* 1–7 (2012).

[25] Call BJ, Goodridge W, Villanueva I, Wan N, Jordan K (2016). Utilizing electroencephalography measurements for comparison of task- specific neural efficiencies: Spatial intelligence tasks. J. Vis. Exp..

[26] Liu CJ (2013). Applying frequency bands to explore the identification of two dimensional figures. Appl. Mech. Mater..

[27] Riečanský I, Katina S (2010). Induced EEG alpha oscillations are related to mental rotation ability: The evidence for neural efficiency and serial processing. Neurosci. Lett..

[28] Gevins A, Smith ME, McEvoy L, Yu D (1997). High-resolution EEG mapping of cortical activation related to working memory: Effects of task difficulty, type of processing, and practice. Cereb. Cortex.

[29] Shah JJ, Woodward J, Smith SM (2013). Applied tests of design skills-part II: Visual thinking. J. Mech. Des..

[30] Oti A, Crilly N (2021). Immersive 3D sketching tools: Implications for visual thinking and communication. Comput. Graph..

[31] Lukačević F, Li S, Becattini N, Škec S (2022). Comparing EEG brain power of mechanical engineers in 3D CAD modelling from 2D and 3D representations. Proc. Des. Soc..

[32] Park, J. A. & Kim, Y. S. Visual reasoning and design processes. *Proc. ICED 2007, 16th Int. Conf. Eng. Des.* **DS 42**, 1–12 (2007).

[33] Ornstein R, Johnstone J, Herron J, Swencionis C (1980). Differential right hemisphere engagement in visuospatial tasks. Neuropsychologia.

[34] Roberts, J. E. & Ann Bell, M. Two- and three-dimensional mental rotation tasks lead to different parietal laterality for men and women. *Int. J. Psychophysiol.* **50**, 235–246 (2003). 10.1016/s0167-8760(03)00195-8 14585492

[35] Gerlič I, Jaušovec N (1999). Multimedia: Differences in cognitive processes observed with EEG. Educ. Technol. Res. Dev..

[36] Fajen, B. R. & Phillips, F. Spatial perception and action. in *Handbook of Spatial Cognition* (eds. Waller, D. & Nadel, L.) 67–80 (American Psychological Association, 2013). 10.1037/13936-004.

[37] Rosso, P., Gopsil, J., Hicks, B. & Burgess, S. Investigating and characterising variability in CAD modelling: An overview. in *Proceedings of CAD’20* 226–230.10.14733/cadconfp.2020.226-230 (2020).

[38] Peirce JW (2019). PsychoPy2: Experiments in behavior made easy. Behav. Res. Methods.

[39] Cohen MX (2017). Where does EEG come from and what does it mean?. Trends Neurosci..

[40] Delorme, A. & Makeig, S. EEGLAB: An open source toolbox for analysis of single-trial EEG dynamics including independent component analysis. *J. Neurosci. Methods* **15**, 9–21 (2004). 10.1016/j.jneumeth.2003.10.009 15102499

[41] Li, S., Becattini, N. & Cascini, G. Correlating design performance to EEG activation: Early evidence from experiental data. in *Proceedings of the Design Society* 771–780. 10.1017/pds.2021.77 (2021).

[42] Jia W, von Wegner F, Zhao M, Zeng Y (2021). Network oscillations imply the highest cognitive workload and lowest cognitive control during idea generation in open-ended creation tasks. Sci. Rep..

[43] De Clercq W, Vergult A, Vanrumste B, Van Paesschen W, Van Huffel S (2006). Canonical correlation analysis applied to remove muscle artifacts from the electroencephalogram. IEEE Trans. Biomed. Eng..

[44] Pfurtscheller, G. & Lopes da Silva, F. H. Event-related EEG/MEG synchronization and desynchronization: basic principles. *Clin. Neurophysiol.* **110**, 1842–1857 (1999). 10.1016/s1388-2457(99)00141-8 10576479

[45] ARTool: Aligned Rank Transform. https://cran.r-project.org/web/packages/ARTool/index.html.

[46] Wobbrock, J. O., Findlater, L., Gergle, D. & Higgins, J. J. The Aligned Rank Transform for nonparametric factorial analyses using only ANOVA procedures. *Conf. Hum. Factors Comput. Syst. Proc.* 143–146. 10.1145/1978942.1978963 (2011).

[47] Willis SG, Wheatley GH, Mitchell OR (1979). Cerebral processing of spatial and verbal-analytic tasks: an EEG study. Neuropsychologia.

[48] Teplan M (2003). Fundamentals of EEG measurment. Meas. Sci. Rev..

